# Stable *qw12-1* Locus Across Environments: High-Resolution QTL Mapping for Sustainable Southern Soybean Crinkle Leaf Disease Resistance Control

**DOI:** 10.3390/plants15071010

**Published:** 2026-03-25

**Authors:** Wenjie Chen, Chunting Zhang, Qian Shi, Xiaohong Guo, Xiayan Qin, Shufang Chen, Kai Sun, Qingyuan Wei, Fuyue Tang, Jiang Liang, Tuanjie Zhao, Yuan Chen

**Affiliations:** 1 Guangxi Crop Genetic Improvement and Biotechnology Laboratory, Cash Crops Research Institute, Guangxi Academy of Agricultural Sciences, Nanning 530007, China; cenwenji1030@163.com (W.C.); kabakebang@126.com (X.G.); qinxiayan045@163.com (X.Q.); xie18074938374@126.com (S.C.); wei4444@yeah.net (Q.W.); tfy0130@163.com (F.T.); liangjiang0626@163.com (J.L.); 2 Key Laboratory of Biology and Genetics Improvement of Soybean, Ministry of Agriculture, Zhongshan Biological Breeding Laboratory (ZSBBL), National Innovation Platform for Soybean Breeding and Industry-Education Integration, State Key Laboratory of Crop Genetics & Germplasm Enhancement and Utilization, College of Agriculture, Nanjing Agricultural University, Nanjing 210095, China; 2021201086l@stu.njau.edu.cn; 3 College of Agriculture, Guangxi University, Nanning 530004, China; 15287493960@163.com; 4 Jiangsu Key Laboratory for Pathogens and Ecosystems, College of Life Sciences, Nanjing Normal University, Nanjing 210023, China; sunkainnu@163.com

**Keywords:** soybean, crinkle leaf disease, recombinant inbred lines, QTL mapping, candidate gene

## Abstract

Severe southern soybean crinkle leaf disease (SSCLD) reduces soybean seed yield by approximately 40%. Identifying the genes that control SSCLD is crucial for breeding resistant varieties and elucidating the molecular mechanisms underlying SSCLD infection. In this study, recombinant inbred lines (RILs, *n* = 236) derived from a cross between Nannong1138-2 (NN1138-2) and Zhengxiaodou (ZXD) were used as experimental materials. A field trial employing a randomized block design was conducted in four environments across two locations, Nanning (2019–2021) and Du’an (2020) in Guangxi, to identify the disease severity grades of SSCLD in the field. QTLs controlling SSCLD were detected via a genetic map constructed using 3255 SLAF (specific locus amplified fragment) markers from the recombinant inbred lines. RT–qPCR was used to analyze candidate gene expression at major effect loci. The results revealed that eight SSCLD-associated QTLs were identified on chromosomes 3, 6, 12, and 17. Notably, the *qw12-1* locus on chromosome 12 was detected across three developmental stages in three of the four environments, explaining 10.18–58.20% of the phenotypic variation. RT–qPCR analysis of 12 disease resistance-related genes within the *qw12-1* interval revealed that *GLYMA_12G233000* and *GLYMA_12G239200* presented significantly higher expression in crinkled leaf lines than in normal leaf lines during the V5 (fifth trifoliolate stage), R2 (full bloom stage), and R6 (full seed stage) stages. These genes were prioritized as potential prime candidates for SSCLD resistance genes. This research provides foundational data for the fine mapping of *qw12-1* and cloning SSCLD-related genes, advancing our understanding of the molecular mechanisms underlying SSCLD.

## 1. Introduction

Soybean (*Glycine max* (L.) Merr.), a globally cultivated legume and a vital source of plant-based protein and unsaturated fatty acids, holds strategic importance in China’s agricultural security. Southern China is a pivotal high-protein soybean production zone [1]. In recent years, severe southern soybean crinkle leaf disease (SSCLD)—a new soybean disease causing wrinkled leaf symptoms resembling those of soybean mosaic virus (SMV)—has occurred in southern China, including areas in Guangxi, Guangdong, Fujian and Guizhou Provinces. Severe SSCLD can cause an approximate 40% reduction in soybean yield, severely impacting local soybean production. Identifying genetic determinants of SSCLD is essential for elucidating its molecular mechanisms and accelerating the development of resistant cultivars.

Perturbations in canonical metabolic pathways during leaf morphogenesis may induce malformed leaf phenotypes. Crinkle leaf disease (CLD), as a distinct foliar deformity, has been documented in cotton (*Gossypium hirsutum* L.) [2], cucumber (*Cucumis sativus* L.) [3], and mulberry (*Morus alba* L.) [4]. Rhizobium sp. strain IC3342 has been reported to trigger leaf crinkling in pigeon pea (*Cajanus cajan* L.) [5,6]. Notably, genetic mutations causing analogous foliar distortions have been observed in plant species such as oilseed rape (*Brassica napus* L.) [7], peach (*Prunus persica* L.) [8], and sesame (*Sesamum indicum* L.) [9,10]. In Arabidopsis thaliana, loss-of-function mutations in the *CRUMPLED LEAF* (*CRL*) gene encoding a plastid envelope protein disrupt cytokinesis, cellular differentiation, and plastid division, resulting in a crumpled leaf phenotype [11,12,13]. In soybean, CLD can be caused by multiple factors, including exogenous triggers such as manganese toxicity [14,15,16], herbicide phytotoxicity [17,18,19], nutritional imbalances [20], and viral infections [21,22,23], or mutations in genes regulating leaf development. For example, mutations in *GLYMA_19G207100* on chromosome 19 of soybean result in crinkled leaves [24]. Additionally, recessive mutations in genes located on chromosomes 7, 8 and 18 have been implicated in crinkled leaf phenotypes [25,26].

In prior research, we confirmed that SSCLD is unrelated to manganese toxicity, agrochemicals, nutrient disorders, or virosis. The evidence strongly implicates soil-borne biotic factors in its pathogenesis [27]. However, the specific biotic factor responsible remains unidentified. The occurrence of SSCLD is likely a symptom resulting from the interaction between soil-borne biotic factors and soybean genes. Identifying the genes controlling SSCLD is crucial for further pinpointing its causative agents. Therefore, identifying genes that govern the soybean’s response to this unknown biotic factor is a rational strategy. It not only provides targets for breeding resistant varieties but can also serve as a critical genetic tool to help identify the pathogen itself, by enabling comparative studies of plant responses in resistant versus susceptible backgrounds. The genetic basis of SSCLD remains poorly understood. In our preliminary work, we mapped a gene, named *CL12*, controlling SSCLD to the end of chromosome 12 in soybean using RNA-seq from high-generation segregating populations [28]. However, potential epistatic loci contributing to SSCLD remain uncharacterized. Specifically, it is unknown whether SSCLD resistance is governed solely by a single major locus like *CL12*, or if it involves a more complex genetic architecture with additional modifying or epistatic QTLs. A comprehensive genetic dissection is therefore necessary to fully understand the resistance mechanism.

In this study, we aimed to comprehensively elucidate the genetic architecture underlying SSCLD. To this end, specific locus amplified fragment sequencing (SLAF-seq) of a recombinant inbred line population composing 236 RILs (designated NJZN-RIL) was used to construct a bin marker-based high-density genetic map. Through years of multi-site and multi-environmental experiments, quantitative trait loci (QTLs) related to SSCLD have been identified. This not only lays the foundation for precise mapping and cloning of the relevant genes, but also prepares for future elucidation of the molecular mechanism underlying SSCLD.

## 2. Results

### 2.1. Leaf Morphological Characteristics of Soybean Under SSCLD

**Figure 1:**
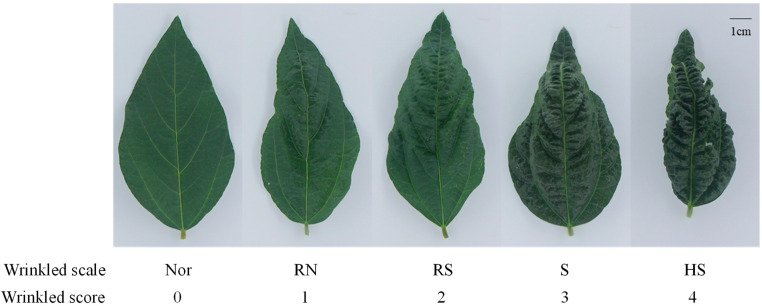
Leaves with different degrees of wrinkling. Nor = normal, RN = Relatively normal, RS = Relatively sensitive, S = sensitive, HS = highly sensitive. Bar = 1cm.

**Figure 2:**
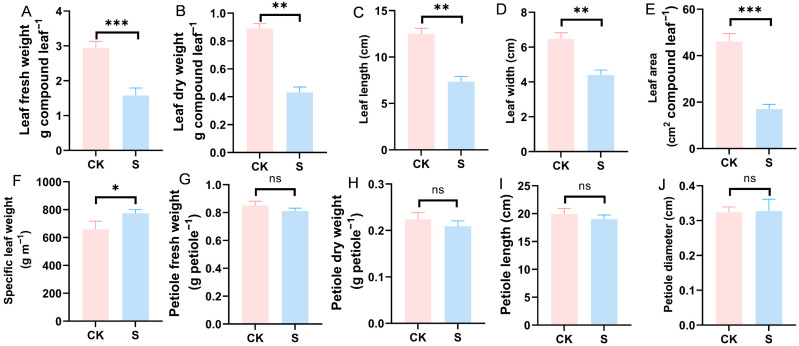
Leaf morphological characteristics of soybean under SSCLD in pot experiment. Treatment S: disease-conducive soil was sterilized by autoclaving at 121 °C for 40 min and then mixed with a microbial-amended substrate at a 15:1 (soil: substrate, *w*/*w*) ratio. Control (CK): non-sterilized disease-conducive soil was mixed with the same substrate at the identical ratio. (**A**) Analyses of leaf fresh weight; (**B**) analyses of leaf dry weight; (**C**) analyses of leaf length; (**D**) analyses of leaf width; (**E**) analyses of leaf area; (**F**) analyses of specific leaf weight; (**G**) analyses of petiole fresh weight; (**H**) analyses of petiole dry weight; (**I**) analyses of petiole length; (**J**) analyses of petiole diameter. *** means *p* < 0.001, ** means *p* < 0.01, * means *p* < 0.05, ns means *p* > 0.05.

A heritable crinkled leaf phenotype (Figure 1) with variable severity was observed in soybeans across Guangxi, Guangdong and Fujian provinces. Severe manifestations included blade curling with apical hooking, sinuous S-shaped venation, and bulliform protrusions along adaxial veins. In the pot experiment, Student’s *t*-tests revealed significant SSCLD-induced morphological aberrations, specifically, reductions in leaf dry weight (*p* = 0.0011), fresh weight (*p* = 0.0004), length (*p* = 0.0012), width (*p* = 0.0011), area (*p* = 0.0002), and specific leaf weight (*p* = 0.037) (Figure 2 and Figure S1).

### 2.2. SSCLD Trait Characterization in the NJZN-RIL Population

**Figure 3:**
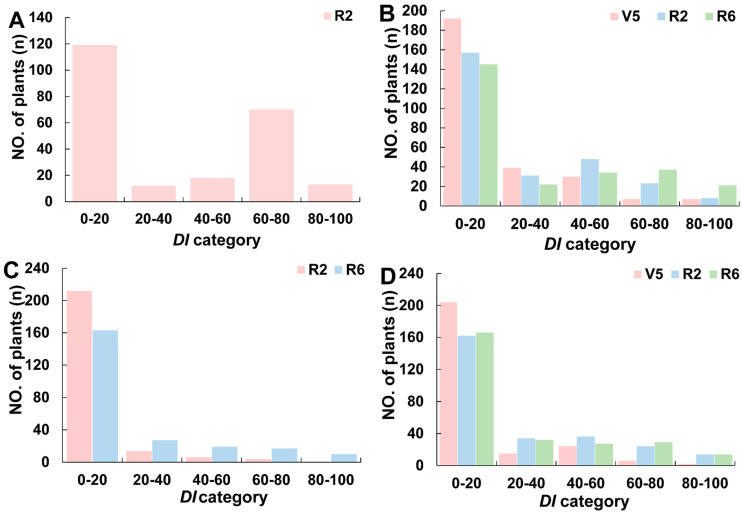
Statistics of leaf DI in NJZN-RIL lines in different periods under four environments. (**A**) In the summer of 2019 in NN; (**B**) in the summer of 2020 in NN; (**C**) in the summer of 2020 in DA; (**D**) in the spring of 2021 in NN. V5 is fifth-node stage, R2 is full bloom stage, and R6 is full seed stage. NN is Nanning, DA is Du’an.

During the analysis of SSCLD resistance in the NJZN-RIL population in the four environments, four lines in the 2019smNN-R2 environment were discarded because the disease severity score of the control material GY_C was less than five; the remaining 232 lines in the environment were ultimately validated (Table 1). In other environments, the disease severity scores of GY_C exceeded five in all cases, and the data of all lines were considered valid. SSCLD exhibited transgressive segregation in the NJZN-RIL populations. Of the two parentals of the RILs, ZXD maintained normal leaf morphology across all four environments (V5, R2, and R6 stages), whereas NN1138-2 displayed mild crinkling solely at 2020smNN–R6. The maximum DI reached 100.00 across all environments except during 2020smDA–R2 (DI = 75.00). Canopy symptom severity peaked at R6 (mean DI = 24.44), followed by R2 and V5. ANOVA revealed highly significant (*p* < 0.01) genotype, environment, and genotype × environment (G × E) effects across developmental stages (Table S2), with broad-sense heritability exceeding 80% (Table 1). The DI distribution in the NJZN-RIL population (Figure 3) exhibited continuous left-skewed patterns across the V5, R2, and R6 stages, with a predominance of accessions receiving scores of 0–20. The proportions ranged from 36.50% (2019smNN-R2) to 80.02% (2020smNN-V5).

Genetic analysis of the secondary segregating populations derived from NN1138-2/ZXD revealed that the parental normal phenotype and F_1_ uniformity were maintained in spring 2024, whereas segregation of the crinkled leaf phenotype was observed in the F_2_ generation in summer 2024 (Table 2). Chi-square tests further confirmed a 3:13 segregation ratio for leaf morphology (R2 stage: χ^2^ = 1.784, *p* = 0.182; R6 stage: χ^2^ = 1.10).

### 2.3. Genetic Linkage Map Construction and QTL Mapping

**Figure 4:**
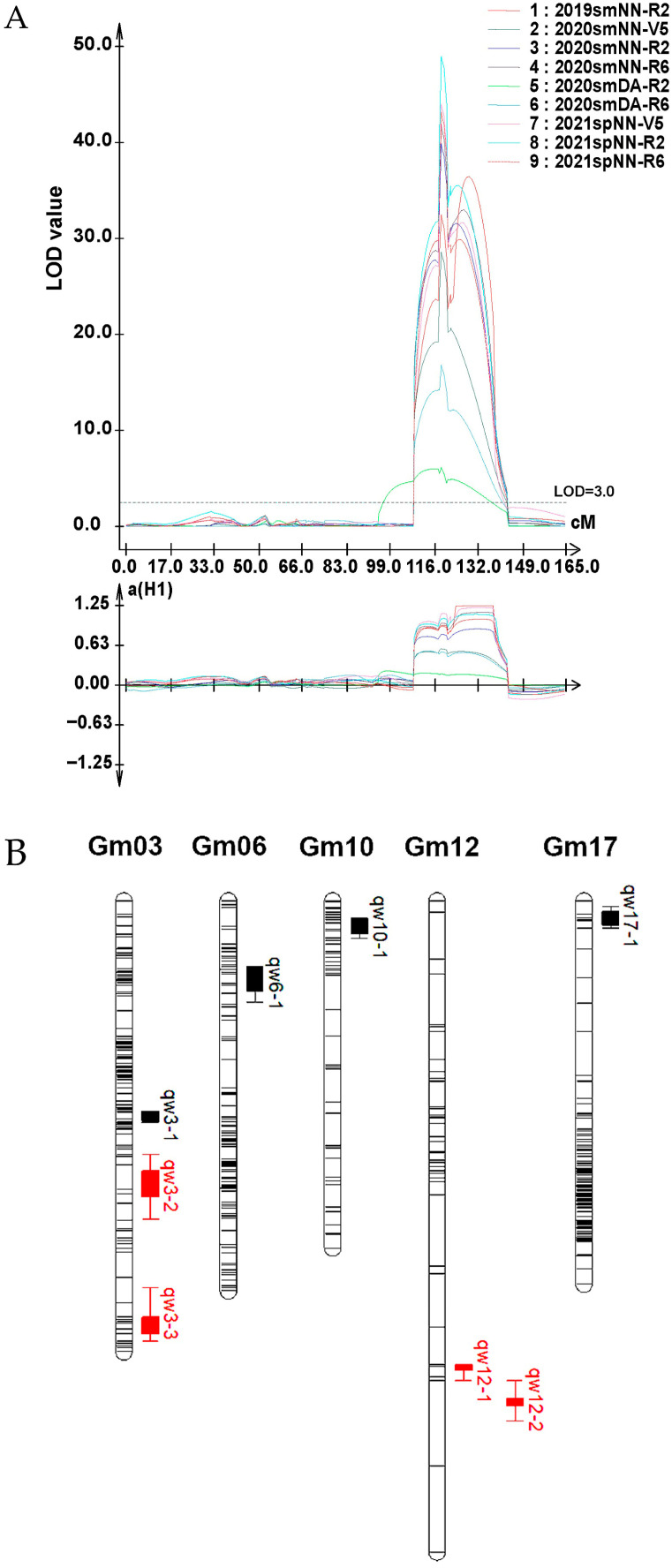
Genetic linkage map construction and QTL mapping for SSCLD. (**A**) QTL for wrinkled-leaf-related traits on chromosome 12 detected by WinQTLcart. 2019smNN is summer 2019 in Nanning, Guangxi Province; 2020smNN is summer 2020 in Nanning, Guangxi Province; 2021spNN is spring 2019 in Nanning, Guangxi Province; 2020smDA is summer 2020 in Du’an Agricultural Sciences. V5 is fifth trifoliolate stage, R2 is full bloom stage, and R6 is full seed stage. The black dashed line indicates the LOD threshold of 3.0 for declaring significant QTL detection. The lower panel displays the additive effect (a(H1)) of the major QTL across these environments, where positive values signify that the heterozygous genotype (H1) confers a resistance advantage, and negative values indicate susceptibility-favoring effects. (**B**) Putative QTLs for leaf shrinkage traits on chromosomes Gm03, Gm06, Gm10, Gm12, and Gm17. The red sites were detected in over two environments, and the black ones in only one. Filled boxes indicate the peak LOD positions of each QTL, and horizontal bars represent the 95% confidence intervals. QTL names (e.g., *qw3-1*) are listed beside the peaks.

As Cao et al. [29] reported, 103,845,237 paired-end reads from the two parents and all lines were mapped to the reference genome Williams 82.a1. Reads mapped to a single locus were considered as effective SLAFs. Consequently, the NJZN-RIL population-based genetic map incorporated 3255 SLAF markers, spanning 20 linkage groups with a total genomic coverage of 2144.85 cM (mean intermarker distance: 0.66 cM) (Table S3). Eight leaf-crinkling-associated QTLs were detected (Figure 4A), with four stable loci (mapped to chromosomes 3 and 12) detected in over two environments. These compounds exhibited additive effects of 0.12–1.25, explaining 5.13–73.67% of the phenotypic variation. A major QTL on chromosome 12, designated as *qw12-1* (Figure 4B), was identified across three developmental stages in four environments. Its additive effects ranged from 0.18 to 1.12, with LOD scores of 6.15–49.02, and it explained 10.18–58.20% of the phenotypic variance (Table S3).

### 2.4. Candidate Gene Analysis for SSCLD

The *qw12-1* locus could be detected at three developmental stages across four environments, so it was selected as the main candidate locus region for SSCLD resistance. Subsequent analysis revealed that, compared to Williams 82.a1 reference genome, Williams 82.a2 exhibited higher integrity and accuracy in sequence assembly within the *qw12-1* region. Therefore, to achieve more precise delimitation of candidate regions and gene prediction, we employed the Williams82.a2 reference genome for further analysis. The flanking markers (Table S4) of *qw12-1* were mapped to the Williams 82.a2 reference genome assembly, defining an interval of approximately 750.88 kb (39,135,606 bp–39,886,428 bp) containing 76 genes, 67 of which have functional annotations (Tables S4 and S5). Given that previous findings suggest that SSCLD may be caused by soil microbial factors [28], we selected 12 disease-resistance-related genes from these 67 genes as candidate genes controlling SSCLD. These genes are: *GLYMA_12G231800*, *GLYMA_12G233000*, *GLYMA_12G233100*, *GLYMA_12G233300*, *GLYMA_12G233600*, *GLYMA_12G233700*, *GLYMA_12G235900*, *GLYMA_12G236500*, *GLYMA_12G238600*, *GLYMA_12G239200*, *GLYMA_12G239700*, and *GLYMA_12G239800*. Among them, *GLYMA_12G231800*, *GLYMA_12G233000*, *GLYMA_12G233100*, and *GLYMA_12G233300*, with similar sequence lengths (12,620 bp to 13,541 bp) and close linkages, encode leucine-rich repeat receptor-like kinases (LRR-RLKs) and may form a functionally related gene cluster. *GLYMA_12G233600*, *GLYMA_12G233700*, *GLYMA_12G235900*, and *GLYMA_12G238600* also encode LRR-RLKs, but with greater distances between members and more variable gene lengths. *GLYMA_12G236500*, *GLYMA_12G239200*, *GLYMA_12G239700*, and *GLYMA_12G239800* encode NB-ARC domain proteins (Table 3 and Table S5).

### 2.5. RT–qPCR Analysis for Candidate Genes

**Figure 5:**
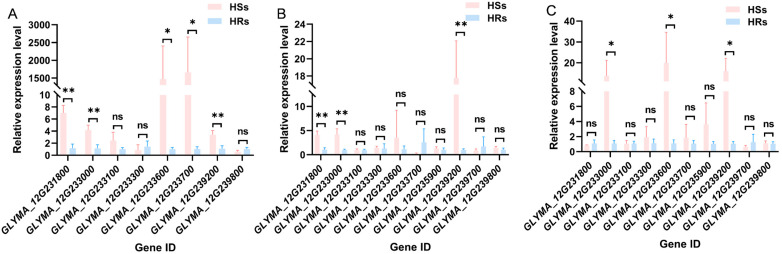
Results of candidate gene RT–qPCR analysis. (**A**), (**B**), and (**C**) represent the V5, R2, and R6 stages of soybean, respectively. HR (high resistance, DI = 0) and HS (high susceptibility, DI > 90) each consist of six independent lines (RILs). For each line, three biological replicates (independent potted plants) were analyzed, and the expression values of the three replicates were averaged to obtain a single value per line. The averaged values of the six HR lines and six HS lines were then compared using Student’s *t*-test. ** means *p* < 0.01, * means *p* < 0.05, ns means *p* > 0.05.

Based on the Williams 82.a2 reference genome information, the CDS sequences of the 12 candidate genes related to SSCLD were downloaded from SoyBase (https://legacy.soybase.org, accessed on 24 January 2022). Molecular markers for RT–qPCR were designed using Primer 5 (Table S6). After gradient PCR to determine the optimal amplification temperature, RT–qPCR was performed on cDNA from young leaves of six normal and crinkled leaf lines in the RIL population (grown in 2022) at the V5, R2, and R6 stages. The results revealed that except for *GLYMA_12G236500* and *GLYMA_12G238600* (no expression in leaves at all three stages) and *GLYMA_12G235900* and *GLYMA_12G239700* (no expression at V5), the other eight genes were expressed. *GLYMA_12G231800* presented significantly greater expression in the crinkled leaves than in the normal leaves at V5 and R2 (*p* < 0.01). *GLYMA_12G233600* was upregulated in the crinkled leaves at V5 and R6 (*p* < 0.05). Notably, *GLYMA_12G233000* and *GLYMA_12G239200* presented significantly greater expression in the crinkled leaves than in the normal leaves across all three growth stages (Figure 5). Therefore, *GLYMA_12G233000* and *GLYMA_12G239200* were preliminarily chosen as SSCLD-related candidate genes.

## 3. Discussion

**Figure 6:**
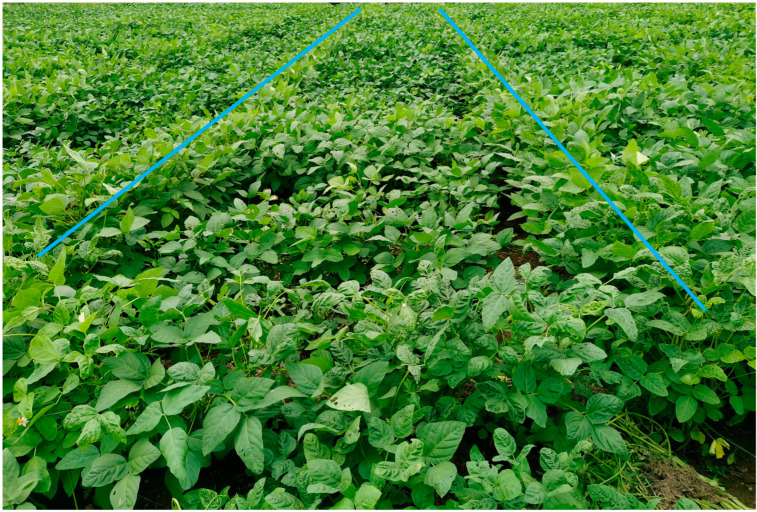
Field evaluation of SSCLD in the RIL population. Blue line indicates the crinkled leaf-susceptible accession GY_C, which was a homozygous crinkle leaf line selected from the F_7_ generation of a cross between GC8 (a normal leaf cultivar) and Y2017-1 (a crinkle-leaf-susceptible line). The region between the two blue lines represents the RIL lines requiring phenotypic evaluation.

Phenotyping accuracy is crucial for gene mapping [30,31]. Uniform treatments enhance phenotyping reliability. For disease resistance assessment, plants were inoculated with specific pathogens, such as Phytophthora sojae for soybean root rot [32,33], Xanthomonas campestris pv. campestris for bacterial spot [34,35], or soybean mosaic virus for soybean mosaic disease [21,22,23]. For the assessment of abiotic stress tolerance, specific stress treatments were applied, such as 200 μmol/L AlCl_3_ for aluminum toxicity [36] or 150 mmol/L NaCl for salt tolerance [37]. Given that the cause of SSCLD is unknown, artificial induction was not possible. To improve SSCLD phenotyping in NJZN-RIL under field conditions, susceptible material GY_C was planted at both ends of each row (Figure 6). Only rows where GY_C showed severe symptoms were retained for analysis, enhancing the reliability of the data. However, the current method which uses a wrinkle index may misclassify heterozygous lines as homozygous. To address this, lines with both normal and wrinkled leaves among single plants should be recorded as heterozygous.

Traits controlled by multiple genes often show transgressive segregation in offspring due to recombination, as observed for traits such as 100-seed weight [38,39,40], plant height [29,41], and protein content [42,43]. Both the NJZN-RIL and the F_2_ populations derived from NN1138-2 and ZXD showed transgressive segregation of SSCLD symptoms, indicating polygenic control (Figure S2). Chi-square analysis of the F_2_ population further supported this, revealing a 3:13 segregation ratio of crinkled to normal leaves (χ^2^ = 1.784, *p* = 0.182). To explain the observed segregation patterns and the environment-dependent phenotype of NN1138-2, we propose a testable genetic model. Assuming that NN1138-2 has the genotype RRAA, with RR and AA representing independent loci where AA is a dominant locus for SSCLD and RR has a dominant epistatic effect, and ZXD has the genotype rraa, then in environments with weak crinkling factors, R can suppress A, resulting in normal leaves, whereas in severe environments with severe crinkling factors, R can only slightly suppress A, causing mild leaf crinkling. This would explain why NN1138-2 exhibited mild crinkling at R6 in the 2020smNN environment, but normal leaves otherwise (Table 1). In the F_2_ population, plants with R and A (9/16 of the F_2_ population) would display normal leaves, leading to a 3:13 segregation ratio. In 2024, a SSCLD resistance assessment of the F_2_ population from NN1138-2 and ZXD revealed 59 crinkled and 133 normal individuals, with a 3:13 ratio (χ^2^ = 1.784, *p* = 0.182). Thus, SSCLD in NN1138-2 and ZXD likely involves two major genes with epistatic effects and multiple minor genes.

Over two years and in four environments, eight QTLs for SSCLD were identified on chromosomes 3, 6, 10, 12, and 17. Some QTLs were detected in specific environments, whereas *qw3-2* and *qw3-3* on chromosome 3 and *qw12-2* on chromosome 12 were detected in multiple environments. Notably, *qw12-1* on chromosome 12 was detected across all four environments and explained 10.18–58.20% of the variation in the crinkled leaf phenotype (Table S3). The QTLs on chromosome 3 had negative additive effects, whereas those on chromosome 12 had positive effects (Table S3). These findings suggest that *qw12-1* might be the AA locus and chromosome 3 QTLs might be the RR locus with epistatic effects. Further studies with F_2_ populations are needed to confirm the epistatic relationship between chromosomes 3 and 12. A line exhibiting severe leaf crinkling will be selected from the NJZN-RIL population and crossed with both parental lines, NN1138-2 and ZXD. The segregation ratio of crinkled leaf individuals will be used to infer the effects of the two loci on the phenotype. Meanwhile, the BSA-seq strategy will be employed to map the chromosomal position of the RR locus.

To date, the cause of SSCLD remains unknown. On the basis of our preliminary studies, we hypothesize that SSCLD symptoms result from interactions between soil-derived microbial factors and soybean susceptibility genes. Genes controlling SSCLD may be related to plant disease resistance. Within the *qw12-1* locus, 12 genes were annotated as potential disease resistance candidates; however, only *GLYMA_12G233000* and *GLYMA_12G239200* showed significantly higher expression in crinkled leaf progenies than in normal leaf lines in disease-infested soil. *GLYMA_12G233000* encodes LRR-RLKs. Plant LRR-RLKs, which are transmembrane protein kinases that perceive external stimuli on the cell surface for signal transduction, can be classified into three categories according to their metabolic functions: growth-related, defense-related, and abiotic stress-resistant [44,45]. Defense-related LRR-RLKs mainly initiate defense responses by recognizing highly conserved pathogen-associated molecular structures, such as bacterial flagellin and fungal chitin, via their extracellular domains [46,47,48], and they can also recognize pathogen-derived fragments or effectors encoded by nonpathogenic genes to regulate immunity [49,50,51]. LRR-RLKs play a significant role in plants’ regulatory networks responding to environmental stress and disease resistance. Although 467 LRR-RLK genes have been identified in soybeans [52], most of their functions remain unverified. The findings of this study hold important implications for further elucidation of the roles of genes encoding LRR-RLKs within the regulatory network of soybean “crinkling factors” inducing crinkle leaf disease.

*GLYMA_12G239200* encodes a nucleotide binding site–leucine-rich repeat domain receptor (NLRs). NLR proteins are associated with effector-triggered immunity (ETI) in plants [53]. These proteins recognize pathogen-derived effector proteins to activate plant resistance responses [54]. To defend against virus infection, NLRs activate the phytohormone-mediated signaling pathways, including salicylic acid (SA), abscisic acid (ABA) and jasmonic acid (JA). In contrast, NLRs conferring resistance to bacteria primarily activate the SA pathway [55]. According to the specificity of their N-terminal domains, NLR proteins are primarily classified into two categories: TNLs (TIR-NBS-LRR) and CNLs (CC-NBS-LRR) [56]. TIR-NBS-LRR genes are widespread in dicotyledonous plants but relatively rare in monocotyledonous species [57]. TNL receptors can trigger immune responses, including reactive oxygen species (ROS) bursts, calcium ion (Ca^2+^) influx, and mitogen-activated protein kinase (MAPK) cascades [58,59]. *GLYMA_12G239200* is classified as a TNL-type NLR protein. The role of *GLYMA_12G239200* in SSCLD development requires further investigation.

In Arabidopsis, mutations in the CRUMPLED LEAF (CRL) gene that encodes a plastid outer envelope membrane protein alter the protein’s structure, disrupt cell division and differentiation, and ultimately result in virus-like leaf crumpling [11,12,13,60]. The overexpression of GmFILa, a FIL subfamily YABBY transcription factor, induces leaf curling in Arabidopsis [61]. Similarly, PtoCYCD3;3 overexpression triggers leaf crinkling in Populus trichocarpa [62]. In soybean, Song et al. [25] identified a crinkly leaf-inducing mutation in a gene named CRINKLY LEAF on chromosome 5, whereas Wang et al. [26] mapped two recessive mutant genes that control virus-like leaf wrinkling, *rl1* and *rl2*, on chromosomes 18 and 8, respectively, noting their homologous nature. Our previous study revealed that the dominant gene *CL12* controls SSCLD. It is located in 1.47 Mb intervals on chromosome 12, with physical positions spanning 39,231,651–40,705,115 bp in the Williams 82.a4 assembly, corresponding to 37,784,970–39,265,045 bp in the Williams 82.a2 assembly. RT–qPCR analysis revealed five candidate genes, namely *GLYMA_12G231800* and *GLYMA_12G233000* [27]. This investigation, employing a 236-line RIL population, identified eight QTLs across chromosomes 3, 6, 10, 12, and 17 over two years across four environments. The *qw12-1* locus on chromosome 12 was consistently detected in all three developmental stages across the four environments, with an interval of approximately 750.88 kb (39,135,606 bp–39,886,428 bp). Two genes, *GLYMA_12G233000* and *GLYMA_12G239200*, were significantly more highly expressed in the crinkled leaf progenies during the V5, R2, and R6 stages. Physically, *CL12* and *qw12-1* share a partially overlapping genomic interval, and the candidate genes for both include *GLYMA_12G233000*. Whether *CL12* and *qw12-1* correspond to the same locus requires further validation.

It is important to note a limitation of the current study. Our gene expression analysis compared asymptomatic (HR, DI = 0) and symptomatic (HS, DI > 90) lines grown under disease-conducive conditions. While this design effectively identified genes whose expression is strongly associated with disease susceptibility, it cannot definitively distinguish whether the observed upregulation in HS lines is a cause of susceptibility (e.g., a dysfunctional defense response) or a consequence of pathogen infection and tissue damage. For instance, the expression of certain genes may be altered as a secondary effect of the pathogen’s manipulation of host physiology or due to the plant’s general stress response to severe infection. Future studies employing time-course sampling of the same genotypes immediately before and after pathogen inoculation, or comparing the same susceptible lines grown in sterilized versus pathogen-infested soil, would be invaluable for elucidating the temporal dynamics and causal relationships of these expression changes. Nevertheless, the strong and consistent association of the candidate gene with the susceptible phenotype across multiple growth stages supports its potential role in the disease response network.

## 4. Materials and Methods

### 4.1. Plant Materials

The NJZN recombinant inbred line (RIL) population, consisting of 236 lines, was derived from a cross between the high-yielding cultivar NN1138-2 and the high-protein landrace ZXD. Although both parental lines exhibit resistance to soybean crinkle leaf disease (SSCLD), we observed severe SSCLD susceptibility in certain progeny when developing high-protein, high-yield cultivars by crossing NN1138-2 and ZXD. To investigate the genetic basis of this resistance breakdown, NJZN-RIL was developed from these two parents. This RIL population was developed through seven cycles of single-seed descent (SSD) from the F_2_ generation at Jiangpu Experimental Station of Nanjing Agricultural University in Nanjing, Jiangsu Province, China. Additionally, F_1_ and F_2_ populations from the same cross were included. To monitor the suitability of field conditions for assessing soybean crinkle leaf and defoliation (SSCLD) severity, the control line GY_C was employed. GY_C is a homozygous crinkle leaf line selected from the F_7_ generation of a cross between GC8 (a normal leaf cultivar) and Y2017-1 (a crinkle-leaf-susceptible line). It was derived from continuous phenotypic selection for the crinkle leaf trait from the F_2_ to F_7_ generations. Notably, under disease pressure, GY_C exhibits greater sensitivity to SSCLD than its susceptible parent Y2017-1 [63].

### 4.2. Experimental Design

Field identification trials of SSCLD in the RIL population were conducted in four environments: Mingyang Research Base of Guangxi Academy of Agricultural Sciences (22°36′30″ N, 108°14′11″ E, 105.64 m altitude, red soil, soil pH 6.2), Nanning, Guangxi Province, in summer 2019, summer 2020, and spring 2021 (2019smNN, 2020smNN, 2021spNN), and Du’an Agricultural Sciences (24°1′7.622″ N, 108°4′30.252″ E, 219.54 m altitude, red soil, soil pH 5.9) in summer 2020 (2020smDA). A randomized block design was used, with two replicates for the 2019smNN trial and three for the others. Each plot was 200 cm long and 40 cm wide, with one row of soybeans planted per plot in hills at 20 cm intervals, with two plants per hill. As the cause of SSCLD-induced leaf crinkling is unknown, plants of the susceptible GY_C material were planted at the beginning and end of each row as a control (Figure 6). Only data from plots where the GY_C control exhibited a disease index (DI) ≥ 60 (confirming a disease-conducive environment) were retained for genetic analysis.

Field evaluation of the crinkled leaf phenotype in the F_1_ and F_2_ populations derived from a cross between NN1138-2 and ZXD was conducted in 2024 at the Mingyang Base of the Guangxi Academy of Agricultural Sciences. The susceptible cultivar GY_C was used as a control and planted alternately after every two rows of the test materials. DI was assessed for each individual plant in the F_1_ and F_2_ populations. Only data from test rows adjacent to GY_C check rows with DI > 60 were included in the analysis.

A pot experiment was conducted using the severely wrinkled leaf line ZN134, derived from the RIL population, to evaluate the effects of SSCLD on soybean leaf morphology. Since previous studies have shown that steam sterilization of disease-conducive soil eliminates its pathogenicity [64], sterilized soil was included as a control treatment. Two soil treatments were prepared: (1) Treatment S: disease-conducive soil was sterilized by autoclaving at 121 °C for 40 min and then mixed with a microbial-amended substrate at a 15:1 (soil: substrate, *w*/*w*) ratio. (2) Control (CK): non-sterilized disease-conducive soil was mixed with the same substrate at the identical ratio. The soil mixtures were placed into pots (top diameter × bottom diameter × height = 215 mm × 173 mm × 148 mm), with three soybean plants per pot. Six biological replicates were established for each treatment group.

### 4.3. Phenotypic Data Collection

The severity of soybean leaf crinkling is classified into five levels (Figure 1). Level 0: Normal, with fully expanded canopy leaves and no crinkling. Level 1: Relatively normal, with canopy leaves showing slight crinkling but no “hooking” and remaining expanded. Level 2: Relatively sensitive, with obvious crinkling and slight “hooking” in canopy leaves. Level 3: Sensitive, with severe crinkling and clear “hooking” in canopy leaves. Level 4: Highly sensitive, with extremely severe crinkling and pronounced “hooking”, with leaves bent downward, unable to open normally.

The severity of leaf crinkling was evaluated for the RIL population and its parents at the fifth trifoliolate stage (V5, fifth nodes on the main stem beginning with unifoliolate node), full bloom stage (R2, flower at node immediately below the uppermost node with a completely unrolled leaf), and full seed stage (R6, pod containing full size green beans at one of the four uppermost nodes with a completely unrolled leaf) [65], and for F_1_ and F_2_ individuals at the R2 stage. The disease index (DI) for each plant or treatment was calculated using the formula:

$$DI\left(\%\right)=\sum \frac{x_{i}n_{i}}{4N}\times 100,$$

where $x_{i}$ represents the crinkling level, $n_{i}$ represents the number of plants with that level, and *N* represents the total number of plants. The disease susceptibility levels were classified as follows: DI = 0, high resistance (HR); 0 < *DI* ≤ 30, resistance (R); 30 < *DI* ≤ 60, middle resistance (MR); 60 < *DI* ≤ 90, sensitivity (S); and *DI* > 90, high sensitivity (HS).

At the R2 stage, the third most basal trifoliate leaf from each pot was taken. Petiole length (PL) was measured with a ruler, and petiole diameter (PD) was measured with a Vernier caliper. The fresh weight of the leaves (LFW) and petioles (PFW) were measured with a balance. Leaves were scanned using an HP LaserJet MFP M227, and leaf length (LL), width (LW), and area (LA) were calculated with ImageJ 2016 [66]. The scanned leaves and petioles were placed in envelopes, deactivated in an oven at 108°C for 30 min, and then dried at 80 °C to a constant weight. The leaf dry weight (LDW) and petiole dry weight (PDW) were measured, and the specific leaf weight (SLW) was calculated with the formula: SLW = LDW/LA.

### 4.4. Genetic Linkage Map Construction

The genotyping of two parents and individuals from the RIL population, along with linkage map construction, was carried out following the methodology described by Cao et al. [29]. Specifically, the Williams 82.a1 reference genome was employed to maintain consistency with the genomic resources used in the initial mapping phase of our study, and SLAF-seq technology [67] was utilized to develop polymorphic markers in the NJZN-RIL population, yielding 3255 SLAF markers. A high-density genetic map was generated using Highmap software [68], with genetic distances between markers calculated via the Kosambi function [69]. MapChart v2.2 was used to draw the linkage map [70]. “Gm” was used for chromosome designation. For example, the first chromosome would be labeled Gm01.

### 4.5. QTL Mapping

Quantitative trait locus (QTL) mapping for crinkle leaf grade was conducted on the NJZN-RIL population across four environments and at three developmental stages, using the composite interval mapping (CIM) method in WinQTLCart 2.5 [71]. The analysis parameters were set as follows: a window size of 10 cM, a walk speed of 1 cM, and 1000 permutation tests at a significance level of *p* < 0.05 to establish the logarithm of odds (LOD) threshold for declaring whether a QTL in a certain chromosomal region was significantly associated with the target trait [72]. Two peaks on the same chromosome were considered separate QTLs if their peak positions were more than 20 cM apart [73]. QTLs detected in at least two environments were considered stable. The identified QTLs were named following standard nomenclature: a lowercase italicized “q” followed by the trait abbreviation “w”, the chromosome number, and a serial number (e.g., qw01.1).

### 4.6. Candidate Gene Screening and RT–qPCR Validation

Major QTLs detected across multiple environments were selected for candidate gene analysis. QTL intervals were initially defined based on the Williams 82.a1 reference genome used for mapping. However, to achieve more precise annotation, the flanking markers of the key QTLs were re-mapped to the newer and more accurate Williams 82.a2 genome assembly for subsequent gene annotation and identification. In summer 2022, six crinkled leaf lines (HSs) and six normal leaf lines (HRs) were grown at the Mingyang Research Base. The total RNA of young leaves was extracted at the V5, R2, and R6 stages using the RNAsimple Total RNA Kit (Tiangen Biochemical, Beijing, China), and cDNA was synthesized via the FastKing RT Kit (with gDNase) (Tiangen Biochemical Technology (Beijing) Co., Ltd.). Actin (forward primer: 5′-GGTGGTTCTATCTTGGCATC-3′, reverse primer: 5′-CTTTCGCTTCAATAACCCTA-3′) was used as the reference gene for RT–qPCR validation of candidate genes, which was performed using two × Cham QTM Universal SYBR qPCR Master Mix (Nanjing NovoZym Biosciences Co., Ltd., Nanjing, China), according to the kit instructions. The qPCR program is detailed in Table S1.

### 4.7. Statistical Analysis

Each experiment was conducted with a minimum of three biological replicates. The leaf morphological indices’ means and standard deviations were calculated in Excel 2019, along with the RIL population’s disease index means, standard deviations, kurtosis, skewness, and coefficients of variation across stages. A Chi-square test was performed to assess whether the observed progeny segregation ratio conformed to the theoretical expectation. Student’s *t*-test was used to determine the significance of differences in the leaf morphological indices and relative express level of candidate genes between diseased and healthy plants. The SAS PROC UNIVARIATE, PROC GLM module was used for ANOVA and broad-sense heritability ($H^{2}$) calculations by SAS Studio (SAS Institute, 2010, Inc., Cary, NC, USA). The broad-sense heritability for DI was estimated using the following equation:

$$H^{2}=\sigma_{g}^{2}/(\sigma_{g}^{2}+\sigma_{ge}^{2}/n+\sigma_{e}^{2}/nr),$$

where $\sigma_{g}^{2}$ is the genotypic variance, $\sigma_{ge}^{2}$ is the genotype-environment interaction variance, $\sigma_{e}^{2}$ is the error variance, *n* is the number of environments, and *r* is the number of replications within an environment [74].

## 5. Conclusions

Severe SSCLD can cause significant reduction in soybean yield, and the genes controlling SSCLD resistance remain unclear. This study identified eight QTLs controlling SSCLD through a three-year, two-location evaluation of NN1138-2 and ZXD parental lines and their derived RIL population across four environments. Among these, the major QTL *qw12-1* on chromosome 12 was consistently detected across all four environments and three developmental stages. RT–qPCR analysis prioritized *GLYMA_12G233000* and *GLYMA_12G239200* within *qw12-1* as candidate genes governing SSCLD. The major QTL locus identified here lays the groundwork for cloning SSCLD-associated genes, unravelling the molecular mechanisms underlying SSCLD, and providing foundational data for breeding SSCLD-resistant soybean cultivars.

## Figures and Tables

**Table 1:** The phenotype of leaf DI of the NJZN-RIL population and its parents.

Period ^a^	Env ^b^	Parental Line DI		RIL Population DI					CV (%) ^c^	H^2^ (%) ^d^
		NN1138-2	ZXD	Mean	SD	Range	Skewness	Kurtosis		
V5	2020smNN	0	0	17.82	22.71	0–100	1.37	1.36	78.47	82.59
	2021spNN	0	0	11.2	19.24	0–100	1.91	3.28	58.21	
	Mean	0	0	14.51	-	-	-	-	-	
R2	2019smNN	0	0	30.93	34.77	0–100	0.39	−1.62	88.96	81.74
	2020smNN	0	0	24.63	27.86	0–100	0.77	−0.61	88.41	
	2020smDA	0	0	4.83	13.69	0–75.00	3.17	9.87	35.28	
	2021spNN	0	0	25.63	28.72	0–100	0.83	−0.58	89.24	
	Mean	0	0	21.51	-	-	-	-	-	
R6	2020smNN	8.33	0	30.23	33.3	0–100	0.57	−1.18	90.78	87.75
	2020smDA	0	0	17.38	26.74	0–100	0.9	−0.53	65	
	2021spNN	0	0	25.72	30.01	0–100	1.44	−0.89	85.7	
	Mean	2.78	0	24.44	-	-	-	-	-	

Note: ^a^ is soybean growth stage, ^b^ is environment, ^c^ is coefficient of variation, ^d^ is broad-sense heritability. 2019smNN is summer 2019 in Nanning, Guangxi Province; 2020smNN is summer 2020 in Nanning, Guangxi Province; 2021spNN is spring 2019 in Nanning, Guangxi Province; 2020smDA is summer 2020 in Du’an Agricultural Sciences. V5 is fifth trifoliolate stage, R2 is full bloom stage, and R6 is full seed stage.

**Table 2:** Statistical analysis of SSCLD resistance rating in parents, F_1_, F_2_ and NJZN-RIL populations grown in different environments.

Materials	Env and Period ^a^	Crinkle Leaf Plants					Normal Leaf Plants	Total	χ^2 b^	df ^c^	*p* Value ^d^
		1	3	5	7	Subtotal	0				
NN1138-2	2024spNN-R2, R6	0	0	0	0	0	50	50	-	-	-
ZXD	2024spNN-R2, R6	0	0	0	0	0	50	50	-	-	-
F_1_	2024spNN-R2, R6	0	0	0	0	0	25	25	-	-	-
F_2_	2024smNN-R2	4	12	36	7	59	314	373	1.784 (N:C 3:13)	1	0.182
	2024smNN-R6	6	13	38	5	62	311	373	1.109 (N:C 3:13)	1	0.311

Note: ^a^ is environment and soybean growth stage, ^b^ is Chi-square test (N, normal leaf plants; C, crinkle leaf plants), ^c^ is the degree of freedom alongside the Chi-square values, ^d^ is the *p* value of the Chi-square test. 2024smNN is summer 2024 in Nanning, Guangxi Province; 2024spNN is spring 2024 in Nanning, Guangxi Province. R2 is full bloom stage, R6 is full seed stage.

**Table 3:** Annotation information and qPCR primers for the 12 candidate genes.

Gene ID	Function	qPCR Forward Primer	qPCR Reverse Primer
*GLYMA_12G231800*	Protein tyrosine kinase	AATAACACTACTTGCCATGTGACAG	CGATCCCAAAAGTGACAGAG
*GLYMA_12G233000*	Leucine-rich repeat	AATAACACTACTTGCCATGTGACAT	CGATTCCCAAAAGTGACAGAT
*GLYMA_12G233100*	Protein tyrosine kinase	ATTAGCAACACTGGTCACTT	GGTCTTATCAAGGCATTATCG
*GLYMA_12G233300*	Protein tyrosine kinase	TAATAGACCTGGTAGATGAGAG	TATTCGTTGGAGACACTTGT
*GLYMA_12G233600*	Leucine-rich repeat protein	GCACGATACCTGAAGCATTA	CTCTAGTTGGCATACCTGAC
*GLYMA_12G233700*	Leucine-rich repeat	ATTCTTGACGGATGCTTCTC	TTGTTCCTTGATGCGGTTAA
*GLYMA_12G235900*	Protein tyrosine kinase	ACAGATACCTATGTGCTACTC	TGAAGGACATGGTGAATTGA
*GLYMA_12G236500*	NB-ARC domain	GATGAACTACGAGACCTGAA	AACACCAGAGGCTTATCAAT
*GLYMA_12G238600*	Leucine-rich repeat	CTAAGTCCGTGTCCAGAATA	CGTCATAGAGTTACCATCCA
*GLYMA_12G239200*	NB-ARC domain	GGATTGGTCTAGGAAGTGTAA	TTCTTCTCTTGGTTCTGCTT
*GLYMA_12G239* *7* *00*	NB-ARC domain	GATACCGTCCTCATCTTAGAA	CCATCTCAGATAGAAGTGTCT
*GLYMA_12G239* *8* *00*	NB-ARC domain	CTCTACCTATGAGGCAAGTTC	GACCGTATTCCTTCTTATGTTG

## Data Availability

The datasets used and/or analyzed during the current study are available from the corresponding author upon reasonable request.

## Supplementary Materials

The following supporting information can be downloaded at: https://www.mdpi.com/article/10.3390/plants15071010/s1, Figure S1: A pot experiment was conducted using the severely wrinkled leaf line ZN134, derived from the RIL population, to evaluate the effects of SSCLD on soybean leaf morphology. S: Disease-conducive soil was sterilized by autoclaving at 121 °C for 40 min and then mixed with a microbial-amended substrate at a 15:1 (soil: substrate, w/w) ratio; CK: Non-sterilized disease-conducive soil was mixed with the same substrate at the identical ratio; Figure S2: The genetic hypothesis underlying the control of the SSCLD gene in NN1138-2 and ZXD. SSCLD may be controlled by two genes at two separate loci. ‘A’ represents the dominant gene for wrinkled leaves, and ‘a’ represents the recessive gene for normal leaves. ‘R’ represents a dominant gene that can inhibit the function of the ‘A’ gene. ‘r’ represents the recessive gene that can inhibit the function of the ‘A’ gene. ‘X’ represents any allele at the gene locus; Table S1: qPCR thermocycling protocol; Table S2: Variance analysis table of phenotypic data; Table S3: QTL analysis for wrinkled leaf trait; Table S4: Annotated gene information in the *qw12-1* interval excepted 12 candidate genes; Table S5: Annotated candidate genes information in the *qw12-1* interval; Table S6: Candidate genes markers for RT-qPCR analysis.

## References

[1] Qin P., Song W., Yang X., Sun S., Zhao X., Yang R., Li N., Hou W., Wu C., Han T. (2014). Regional distribution of protein and oil compositions of soybean cultivars in China. Crop Sci..

[2] Sattar M.N., Kvarnheden A., Saeed M., Briddon R.W. (2013). Cotton leaf curl disease—An emerging threat to cotton production worldwide. J. Gen. Virol..

[3] Rudnieva T., Shevchenko T., Shevchenko A., Shevchenko I., Budzanivska (2018). Cucumber green mottle mosaic virus in agroecosystems of Ukraine. Bull. Taras Shevchenko Natl. Univ. Kyiv. Ser. Biol..

[4] Lu Q.-Y., Wu Z.-J., Xia Z.-S., Xie L.-H. (2015). Complete genome sequence of a novel monopartite geminivirus identified in mulberry (*Morus alba* L.). Arch. Virol..

[5] Upadhyaya N.M., Parker C.W., Letham D.S., Scott K.F., Dart P.J. (1991). Evidence for cytokinin involvement in *Rhizobium* (IC3342)-induced leaf curl syndrome of pigeonpea (*Cajanus cajan* Millsp.). Plant Physiol..

[6] Yong J.W.H., Letham D.S., Wong S.C., Farquhar G.D. (2014). Rhizobium-induced elevation in xylem cytokinin delivery in pigeonpea induces changes in shoot development and leaf physiology. Funct. Plant Biol..

[7] Tan Y., Ren L., Wang J., Ran S., Wu L., Chen Z., Qu C., Li J., Liu L. (2023). Identification and characterization of a curly-leaf locus *CL1* encoding an IAA2 protein in *Brassica napus*. Crop J..

[8] Wiedenhoeft A.C., Arévalo R., Ledbetter C., Jakes J.E. (2016). Structure-property characterization of the crinkle-leaf peach wood phenotype: A future model system for wood properties research?. JOM.

[9] Liu H., Zhou F., Zhou T., Yang Y., Zhao Y. (2023). Histological, cytological, and ultrastructural analysis of a novel sesame mutant JQA showing wrinkled leaf and abort anther. J. Plant Growth Regul..

[10] Zhou F., Yang Y., Zhou T., Liu H. (2024). Unveiling the unique phenotypic, photosynthetic, and biochemical traits of the JQA wrinkled leaf mutant in sesame (*Sesamum indicum* L.). J. Plant Growth Regul..

[11] Šimková K., Kim C., Gacek K., Baruah A., Apel K. (2012). The chloroplast division mutant caa33 of *Arabidopsis thaliana* reveals the crucial impact of chloroplast homeostasis on stress acclimation and retrograde plastid-to-nucleus signaling. Plant J..

[12] Asano T., Yoshioka Y., Kurei S., Sakamoto W., Machida Y., Sodmergen (2004). A mutation of the *CRUMPLED LEAF* gene that encodes a protein localized in the outer envelope membrane of plastids affects the pattern of cell division, cell differentiation, and plastid division in Arabidopsis. Plant J..

[13] Chen Y., Asano T., Fujiwara M.T., Yoshida S., Machida Y., Yasushi Y. (2009). Plant cells without detectable plastids are generated in the crumpled leaf mutant of *Arabidopsis thaliana*. Plant Cell Physiol..

[14] Junior J.L., Reis A.R., Rossi M.L., Cabral C.P., Malavolta E. (2010). Changes in the ultrastructure of soybean cultivars in response to manganese supply in solution culture. Sci. Agric..

[15] Reddy M.R.A.A., Ronaghi A., Bryant J.A. (1991). Differential responses of soybean genotypes to excess manganese in an acid soil. Plant Soil..

[16] Santos E.F., Kondo Santini J.M., Paixão A.P., Júnior E.F., Lavres J., Campos M., Reis A.R. (2017). Physiological highlights of manganese toxicity symptoms in soybean plants: Mn toxicity responses. Plant Physiol. Biochem..

[17] Silva D.R.O.D., Silva E.D.N.D., Aguiar A.C.M.D., Novello B.D., Silva A.A.A.D., Basso C.J. (2018). Drift of 2,4-D and dicamba applied to soybean at vegetative and reproductive growth stage. Ciência Rural..

[18] Robinson A.P., Simpson D.M., Johnson W.G. (2013). Response of glyphosate-tolerant soybean yield components to dicamba exposure. Weed Sci..

[19] Kelley K.B., Wax L.M., Hager A.G., Riechers D.E. (2005). Soybean response to plant growth regulator herbicides is affected by other postemergence herbicides. Weed Sci..

[20] Hopkins E.W. (1933). Leaf-wrinkle, a nutritional disorder of soybean. Plant Physiol..

[21] Che Z., Yan H., Liu H., Yang H., Du H., Yang Y., Liu B., Yu D. (2020). Genome-wide association study for soybean mosaic virus SC3 resistance in soybean. Mol. Breed..

[22] Liu Q., Hobbs H.A., Domier L.L. (2019). Genome-wide association study of the seed transmission rate of soybean mosaic virus and associated traits using two diverse population panels. Theor. Appl. Genet..

[23] Zhang Y., Du H., Zhao T., Liao C., Feng T., Qin J., Liu B., Kong F., Che Z., Chen L. (2023). *GmTOC1b* negatively regulates resistance to soybean mosaic virus. Crop J..

[24] Ochar K., Hong S.B., Zhou M.M., Liu Z.X., Gao H.W., Flamlom S., Qiu L.J. (2022). Identification of the genetic locus associated with the crinkled leaf phenotype in a soybean (*Glycine max* L.) mutant by BSA-Seq technology. J. Integr. Agric..

[25] Song X., Wei H., Cheng W., Yang S., Zhao Y., Li X., Luo D., Zhang H., Feng X. (2015). Development of INDEL markers for genetic mapping based on whole genome resequencing in soybean. G3.

[26] Wang Y., Chen W., Zhang Y., Liu M., Kong J., Yu Z., Jaffer A.M., Gai J., Zhao T. (2016). Identification of two duplicated loci controlling a disease-like rugose leaf phenotype in soybean. Crop Sci..

[27] Chen W., Chen Y., Wei Q., Tang F., Guo X., Liang J. (2024). Analysis of factors causing soybean crinkle leaf in southern China soil. Soybean Sci..

[28] Chen W., Chen Y., Wei Q., Tang F., Guo X., Chen S., Qin X., Wei R., Liang J. (2024). Identification of candidate genes controlling SSCLD by utilizing high-generation segregating populations RNA-seq. Sci. Agric. Sin..

[29] Cao Y., Li S., He X., Chang F., Kong J., Gai J., Zhao T. (2017). Mapping QTLs for plant height and flowering time in a Chinese summer planting soybean RIL population. Euphytica.

[30] Sun L., Jiang L., Ye M., Zhu X., Wang J., Gosik K., Wu R., Pontarotti P. (2015). Functional Mapping: How to Map Genes for Phenotypic Plasticity of Development.

[31] Cobb J.N., DeClerck G., Greenberg A., Greenberg A., Clark R., McCouch S. (2013). Next-generation phenotyping: Requirements and strategies for enhancing our understanding of genotype-phenotype relationships and its relevance to crop improvement. Theor. Appl. Genet..

[32] He H., Zhai Q., Tang Y., Gu X., Pan H., Zhang H. (2023). Effective biocontrol of soybean root rot by a novel bacterial strain Bacillus siamensis HT1. Physiol. Mol. Plant Pathol..

[33] Sahoo D.K., Das A., Huang X.Q., Cianzio S., Bhattacharyya M.K. (2021). Tightly linked *Rps12* and *Rps13* genes provide broad-spectrum Phytophthora resistance in soybean. Sci. Rep..

[34] Cardoso-Sichieri R., Oliveira L.S., Lopes-Caitar V.S., Silva D.C.G.D., Lopes I.D.O.N., Oliveira M.F.D., Arias C.A.A., Abdelnoor R.V., Marcelino-Guimarães F.C. (2024). Genome-wide association studies and QTL mapping reveal a new locus associated with resistance to bacterial pustule caused by *Xanthomonas citri* pv. *glycines* in Soybean. Plants.

[35] Capobiango Da Fonseca P., Maria Barbosa R., Ferreira D.D.O., Badel J.L., Schuster I., Vieira R.F., Sliva F.L.D. (2022). Genome-wide association study reveals molecular markers and genes potentially associated with soybean (*Glycine max*) resistance to *Xanthomonas citri* pv. *glycines*. Plant Breed..

[36] Liu G., Li D., Mai H., Lin X., Lu X., Chen K., Wang R., Riaz M., Tian J., Liang C. (2024). *GmSTOP1-3* regulates flavonoid synthesis to reduce ROS accumulation and enhance aluminum tolerance in soybean. J. Hazard. Mater..

[37] Hu J., Zhuang Y., Li X., Li X., Sun C., Ding Z., Xu R., Zhang D. (2022). Time-series transcriptome comparison reveals the gene regulation network under salt stress in soybean (*Glycine max*) roots. BMC Plant Biol..

[38] Luo S., Jia J., Liu R., Wei R., Guo Z., Cai Z., Chen B., Liang F., Xia Q., Nian H. (2023). Identification of major QTLs for soybean seed size and seed weight traits using a RIL population in different environments. Front. Plant Sci..

[39] Kumar R., Saini M., Taku M., Debbarma P., Mahto R.K., Ramlal A., Sharma D., Rajendran A., Pandey R., Gaikwad K. (2023). Identification of quantitative trait loci (QTLs) and candidate genes for seed shape and 100-seed weight in soybean [ *Glycine max* (L.) Merr.]. Front. Plant Sci..

[40] Yang P., Shu C., Chen L., Xu J., Wu J., Liu K. (2012). Identification of a major QTL for silique length and seed weight in oilseed rape (*Brassica napus* L.). Theor. Appl. Genet..

[41] Wang P., Sun X., Zhang K., Fang Y., Wang J., Yang C., Li W., Ning H. (2021). Mapping QTL/QTN and mining candidate genes for plant height and its response to planting densities in soybean [*Glycine max* (L.) Merr.] through a FW-RIL population. Mol. Breed..

[42] Jamison D.R., Chen P., Hettiarachchy N.S., Miller D.M., Shakiba E. (2024). Identification of quantitative trait loci (QTL) for sucrose and protein content in soybean seed. Plants.

[43] Park H.R., Seo J.H., Kang B.K., Kim J.H., Heo S.V., Choi M.S., Ko J.Y., Kim C.S. (2023). QTLs and candidate genes for seed protein content in two recombinant inbred line populations of soybean. Plants.

[44] Yan W., Ni Y., Zhao H., Liu X., Jia M., Zhao X., Li Y., Miao H., Liu H., Zhang H. (2024). Comprehensive analysis of sesame LRR-RLKs: Structure, evolution and dynamic expression profiles under *Macrophomina phaseolina* stress. Front. Plant Sci..

[45] Peng Y., Ming Y., Jiang B., Zhang X., Fu D., Lin Q., Zhang X., Wang Y., Shi Y., Gong Z. (2024). Differential phosphorylation of Ca^2+^-permeable channel CYCLIC NUCLEOTIDE-GATED CHANNEL20 modulates calcium-mediated freezing tolerance in Arabidopsis. Plant Cell..

[46] Coleman A.D., Maroschek J., Raasch L., Takken F.L.W., Ranf S., Hückelhoven R. (2021). The Arabidopsis leucine-rich repeat receptor-like kinase MIK2 is a crucial component of early immune responses to a fungal-derived elicitor. New Phytol..

[47] Li T., Jarquin Bolaños E., Stevens D.M., Sha H., Prigozhin D.M., Coaker G. (2025). Unlocking expanded flagellin perception through rational receptor engineering. Nat. Plants.

[48] Hao Z., Wang T., Chen D., Shen R., Zhang G., Qian Q., Zhu L. (2025). Leucine-rich repeat protein family regulates stress tolerance and development in plants. Rice Sci..

[49] Varshney K., Gutjahr C. (2023). KAI2 can do: Karrikin receptor function in plant development and response to abiotic and biotic factors. Plant Cell Physiol..

[50] Soltabayeva A., Dauletova N., Serik S., Sandybek M., Omondi J.O., Kurmanbayeva A., Srivastava S. (2022). Receptor-like kinases (LRR-RLKs) in response of plants to biotic and abiotic stresses. Plants.

[51] Ali S., Tyagi A., Mir Z.A. (2024). Plant immunity: At the crossroads of pathogen perception and defense response. Plants.

[52] Wang Q., Zhao X., Sun Q., Mou Y., Wang J., Yan C., Yuan C., Li C., Shan S. (2024). Genome-wide identification of the *LRR-RLK* gene family in peanut and functional characterization of AhLRR-RLK265 in salt and drought stresses. Int. J. Biol. Macromol..

[53] Nguyen Q.M., Iswanto A.B.B., Son G.H., Kim S.H. (2021). Recent advances in effector-triggered immunity in plants: New pieces in the puzzle create a different paradigm. Int. J. Mol. Sci..

[54] Wang W., Liu N., Gao C., Rui L., Jiang Q., Chen S., Zhang Q., Zhong G., Tang D. (2021). The truncated TNL receptor TN2-mediated immune responses require ADR1 function. Plant J..

[55] Zhu M., Feng M., Tao X. (2025). NLR-mediated antiviral immunity in plants. J. Integr. Plant Biol..

[56] Liu J., Cheng Y., Ruan M., Ye Q., Wang R., Yao Z., Zhou G., Liu C., Wan H. (2025). Phylogenetic, structural, and evolutionary insights into pepper *NBS-LRR* resistance genes. Int. J. Mol. Sci..

[57] Zhu N., Feng Y., Shi G., Zhang Q., Yuan B., Qiao Q. (2024). Evolutionary analysis of TIR- and non-TIR-NBS-LRR disease resistance genes in wild strawberries. Front. Plant Sci..

[58] Bernoux M., Chen J., Zhang X., Newell K., Hu J., Deslandes L., Dodds P. (2023). Subcellular localization requirements and specificities for plant immune receptor Toll-interleukin-1 receptor signaling. Plant J..

[59] Maruta N., Burdett H., Lim B.Y.J., Hu X., Desa S., Manik M.K.M., Kobe B. (2022). Structural basis of NLR activation and innate immune signalling in plants. Immunogenetics.

[60] Trejo-Saavedra D.L., Vielle-Calzada J.P., Rivera-Bustamante R.F. (2009). The infective cycle of Cabbage leaf curl virus (CaLCuV) is affected by *CRUMPLED LEAF* (*CRL*) gene in *Arabidopsis thaliana*. Virol. J..

[61] Yang H., Shi G., Li X., Hu D., Cui Y., Hou J., Yu D., Huang F. (2019). Overexpression of a soybean *YABBY* gene, *GmFILa*, causes leaf curling in *Arabidopsis thaliana*. BMC Plant Biol..

[62] Guan C., Xue Y., Jiang P., He C., Zhuge X., Lan T., Yang H. (2021). Overexpression of *PtoCYCD3*;*3* promotes growth and causes leaf wrinkle and branch appearance in *Populus*. Int. J. Mol. Sci..

[63] Chen W., Chen Y., Wei Q., Guo X., Tang F., Zhao T., Liang J. (2022). Changes of leaf morphology during the occurrence of wrinkled leaves in southern soybean and its effects on yield traits. J. South. Agric..

[64] Jenkinson D.S., Powlson D.S. (1976). The effects of biocidal treatments on metabolism in soil. V. A method for measuring soil biomass. Soil. Biol. Biochem..

[65] Fehr W.R., Gaviness C.E., Burmood D.T., Pennington J.S. (1971). Stage of development descriptions for soybeans (*Glycine max* L.) Merrill. Crop Sci..

[66] Arena E.T., Rueden C.T., Hiner M.C., Wang S., Eliceiri K.W. (2016). Quantitating the cell: Turning images into numbers with ImageJ. WIREs Dev. Biol..

[67] Sun X., Liu D., Zhang X., Li W., Liu H., Hong W., Jiang C., Guan N., Ma C., Zeng H. (2013). SLAF-seq: An efficient method of large-scale de novo SNP discovery and genotyping using high-throughput sequencing. PLoS ONE.

[68] Liu D., Ma C., Hong W., Huang L., Liu M., Liu H., Zeng H., Deng D., Xin H., Song J. (2014). Construction and analysis of high-density linkage map using high-throughput sequencing data. PLoS ONE.

[69] Kosambi D.D., Ramaswamy R. (2016). The Estimation of Map Distances from Recombination Values.

[70] Voorrips R.E. (2002). MapChart: Software for the Graphical Presentation of Linkage Maps and QTLs. J. Hered..

[71] Wang S., Basten C.J., Zeng Z.B. (2012). Windows QTL Cartographer 2.5.

[72] Churchill G.A. (1994). Empirical threshold values for quantitative trait mapping. Genetics.

[73] Ravi K., Vadez V., Isobe S., Varshney R. (2021). Identification of several small main-effect QTLs and a large number of epistatic QTLs for drought tolerance related traits in groundnut *(Arachis hypogaea* L.). Theor. Appl. Genet..

[74] Nyquist E.W., Baker R.J. (1991). Estimation of heritability and prediction of selection response in plant populations. Crit. Rev. Plant Sci..

