# Supplementary material for: Stable qw12-1 Locus Across Environments: High-Resolution QTL Mapping for Sustainable Southern Soybean Crinkle Leaf Disease Resistance Control
Source: Plants (Basel). 2026 Mar 25;15(7):1010. doi: 10.3390/plants15071010 (PMC13074802; doi:10.3390/plants15071010)
Supplement: Supplementary file 1 [file plants-15-01010-s001.zip › Figure S1-S2.pdf]

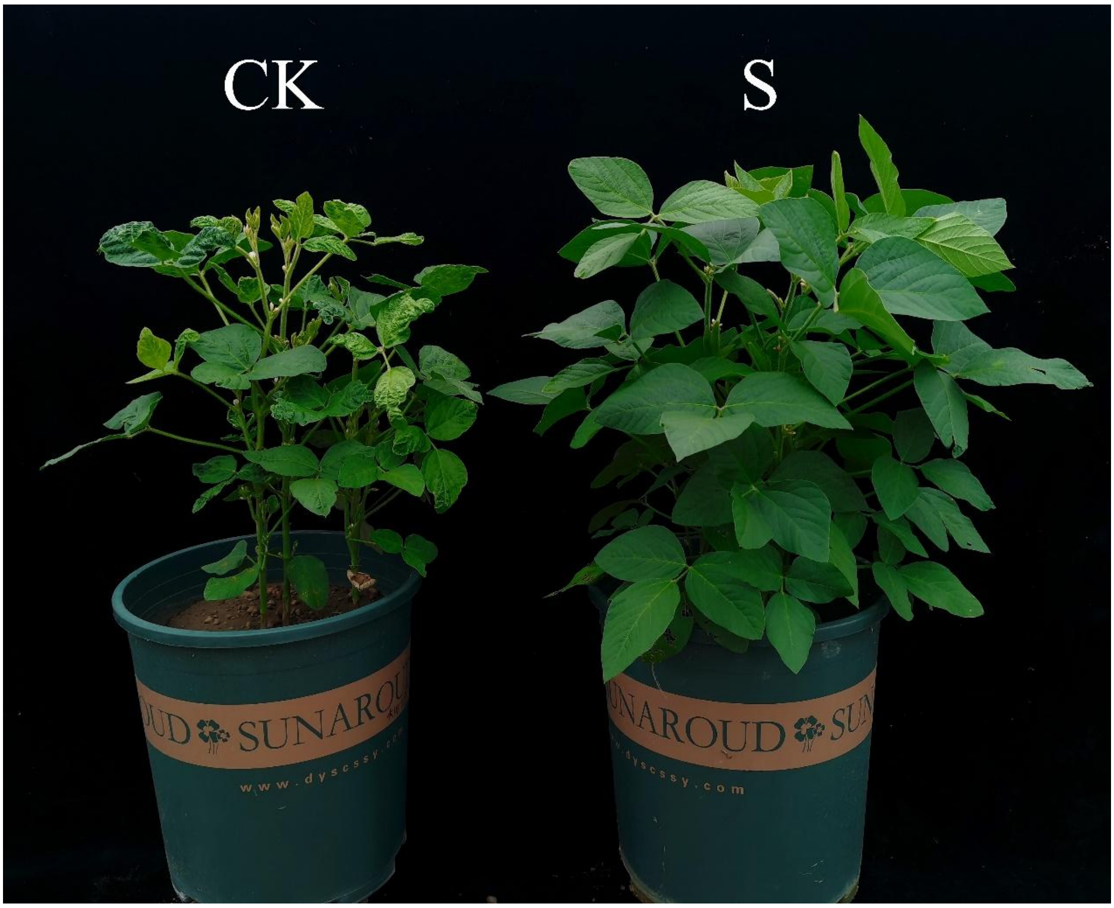

Figure S1. A pot experiment was conducted using the severely wrinkled leaf line ZN134, derived from the RIL population, to evaluate the effects of SSCLD on soybean leaf morphology. S: Disease-conducive soil was sterilized by autoclaving at 121 °C for 40 min and then mixed with a microbial-amended substrate at a 15:1 (soil: substrate, w/w) ratio; CK: Non-sterilized disease-conducive soil was mixed with the same substrate at the identical ratio.

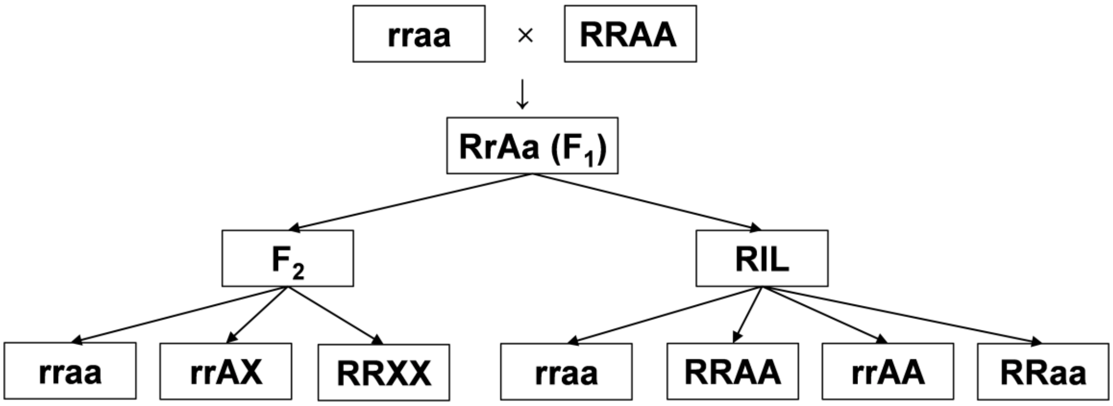

Figure S2. The genetic hypothesis underlying the control of the SSCLD gene in NN1138-2 and ZXD. SSCLD may be controlled by two genes at two separate loci. 'A' represents the dominant gene for wrinkled leaves, and 'a' represents the recessive gene for normal leaves. 'R' represents a dominant gene that can inhibit the function of the 'A' gene. 'r' represents the recessive gene that can inhibit the function of the 'A' gene. 'X' represents any allele at the gene locus.
